# Injectable *PLGA*-based *In Situ* Forming Subconjunctival Implant for Sustained Ocular Delivery of *Ketotifen Fumarate*: Formulation, Drug Release, and Biocompatibility Studies

**DOI:** 10.1208/s12249-025-03142-3

**Published:** 2025-06-02

**Authors:** Nur Mita, Xuejia Kang, Pengyu Chen, Oladiran Fasina, Shenise Howard, Anna-Catherine Bowden, Richard J. McMullen, Amol Suryawanshi, R. Jayachandra Babu

**Affiliations:** 1 Department of Drug Discovery and Development, Harrison College of Pharmacy, Auburn University, Auburn, AL 36849, United States of America; 2 Faculty of Pharmacy, Mulawarman University, Samarinda, Kalimantan Timur 75119, Indonesia; 3 Materials Research and Education Center, Materials Engineering, Department of Mechanical Engineering, Auburn University, Auburn, AL 36849, United States of America; 4 Department of Biosystems Engineering, Samuel Ginn College of Engineering, Auburn University, Auburn, AL 36849, United States of America; 5 Department of Clinical Sciences, College of Veterinary Medicine, Auburn University, Auburn, AL 36849, United States of America; 6 Equine Department, Vetsuisse Faculty, University of Zurich, 8057 Zurich, Switzerland; 7 Department of Population Health and Pathobiology, College of Veterinary Medicine, North Carolina State University, Raleigh, NC, United States of America

**Keywords:** *in situ* forming subconjunctival implant, ketotifen fumarate, PLGA

## Abstract

*Ketotifen fumarate* is very effective in treating ocular conditions like allergic conjunctivitis. However, its clinical effectiveness is limited by rapid washout, frequent dosing, and poor bioavailability. This study developed a *ketotifen fumarate*-loaded *in situ* forming subconjunctival injectable implant (*ISFSI*) for use in large animal species such as horses and alpacas. *ISFSIs* were formulated by dispersing *ketotifen fumarate* in *polylactide-co-glycolide* (*PLGA*) combined with different release retarders (*Kolliphor*^®^ RH 40, *Labrasol*^®^, and *Maisine*^®)^, then characterized for visual appearance, viscosity, solidification time, and *in vitro* drug release. The changes in the release medium pH and the solidified ISFSI wet weight were monitored for up to 4 weeks. The selected *ISFSI*, KFM5, was further characterized by scanning electron microscopy (SEM), differential scanning calorimetry (DSC), and rheological behavior. The injectability of KFM5 was predicted using the power law for five different syringe and needle dimensions. Cytotoxicity was tested using the Alamar Blue assay and Calcein AM/PI staining on a human corneal epithelial cell line (HCE-T). Results showed that all *ISFSI* formulations were clear yellowish, with drug recoveries exceeding 95%, varying viscosities, and solidified upon contact with the release medium. KFM5 exhibited a triphasic drug release profile with the lowest burst release (4.91 ± 0.55%) and followed Higuchi kinetics. SEM imaging revealed a “hollow and sponge-like” structure, while DSC confirmed the crystallinity and thermal transitions of *ketotifen fumarate* within the solidified implant. Rheological analysis indicated shear-thinning fluid behavior with acceptable injection forces. Cytotoxicity assays confirmed > 90% cell viability, confirming biocompatibility in the ocular tissue.

## Introduction

Ocular drug delivery faces significant challenges due to the eye's anatomical and physiological barriers, limiting drug absorption, retention, and therapeutic effectiveness. Major obstacles include rapid tear turnover, blinking, limited corneal permeability, and efflux transporters, all contributing to the poor bioavailability of conventional ophthalmic formulations [1, 2]. Ocular diseases, such as *keratoconjunctivitis*, often require prolonged treatment with topical medications to effectively manage symptoms and prevent complications [3, 4]. However, conventional formulations, such as eye drops, suffer from rapid elimination, requiring multiple daily administrations and leading to inconvenience in veterinary applications and poor patient adherence in human use [1, 2].

*Ketotifen fumarate*, a selective histamine *H*_1_ receptor antagonist with mast cell-stabilizing properties, is commonly used for *allergic conjunctivitis* and inflammatory ocular conditions in humans as well as veterinary animal species [5–8]. It mitigates symptoms like itching, redness, and swelling by inhibiting histamine release and other inflammatory mediators from mast cells and eosinophils while also preventing eosinophil migration to ocular tissues [8–10]. *Ketotifen fumarate* is a Biopharmaceutics Classification System (BCS) class II drug with limited water solubility due to its lipophilic nature (*logP* 3.49). As a weak base, it predominantly exists in its ionized (positively charged) form at physiological *pH*, which affects its solubility, interaction with biological membranes, and overall pharmacokinetics [11, 12]. *Ketotifen fumarate* is available as topical eye drops, oral tablets, and nasal sprays [5, 10, 12, 13]. While *ketotifen* eye drops (0.025%) are widely used, they require frequent administration due to rapid elimination [2, 5]. Oral tablets (1 mg) provide systemic allergy relief but have drawbacks such as delayed onset and side effects like drowsiness and weight gain [7, 14, 15]. Despite therapeutic benefits, these formulations suffer from poor ocular retention, limited corneal permeability (resulting in < 5% ocular bioavailability), and potential systemic side effects, underscoring the need for an ocular drug delivery system for a long-term release of this drug [16, 17].

To address these limitations, various advanced delivery strategies have been investigated to enhance the ocular bioavailability of ketotifen fumarate, including contact lens-mediated systems, nanoparticle carriers, and transdermal formulations. Drug-eluting contact lenses have shown some potential for sustained delivery [18]; however, their effectiveness is constrained by short release durations and external factors such as blinking and tear flow, making them less reliable, particularly in sensitive eyes. Nanoparticle-based systems, including those utilizing Eudragit or PLGA matrices, have demonstrated the ability to prolong drug release [19, 20], yet, when applied topically, they remain susceptible to rapid clearance from the ocular surface, resulting in limited therapeutic benefit. Similarly, transdermal delivery approaches can achieve systemic absorption [21], but they lack the ability to selectively target ocular tissues, potentially leading to suboptimal intraocular drug levels and unwanted systemic side effects. These challenges underscore the need for a localized and sustained-release platform that can bypass precorneal clearance mechanisms and maintain therapeutic concentrations at the site of action.

Moreover, various long-acting injectables and implantable delivery systems, including subconjunctival implants, have been explored to improve ocular drug retention and therapeutic outcomes [17, 22–28]. *In situ* forming implants (*ISFIs*) have emerged as a promising sustained-release platform for ocular drug delivery of various drug molecules [28–34]. The application of this system at a subconjunctival site to form an *in situ* forming subconjunctival implant (*ISFSI*) could overcome the limitations of conventional ocular drug delivery and surgical subconjunctival implant systems since *ISFSIs* are injectable and biodegradable polymeric systems that transition from a liquid dispersion to a solid or semi-solid implant *in vivo* by solvent exchange mechanism, enabling prolonged drug release at the ocular target site [17, 22, 28, 35]. These formulations typically use *poly(lactide-co-glycolide) (PLGA),* a well-established biodegradable polymer that undergoes hydrolytic degradation into non-toxic byproducts [22, 31, 36]. The degradation rate of *PLGA* can be finely tuned by adjusting the lactic acid-to-glycolic acid ratio, allowing for precise control of drug release kinetics [31, 36]. Compared to other biodegradable polymers, *PLGA* provides superior degradation control and mechanical properties, making it highly suitable for ocular applications [22, 37]. The clinical success of *PLGA*-based systems is demonstrated by FDA-approved products such as *Ozurdex*^®^ and *Durysta*^™^, which validate the efficacy of *PLGA* in sustained ocular drug delivery [38, 39].

For *ISFI* formation, the polymer must be dissolved in a suitable solvent, as the choice of solvent plays a crucial role in implant formation, drug release kinetics, and biocompatibility. Solvents such as *N-methyl-2-pyrrolidone (NMP), triacetin, and dimethyl sulfoxide (DMSO)* have been explored for *ISFI* formulations [31, 37]. Among them, *DMSO* offers several distinct advantages for ocular drug delivery since in addition to its high water-miscibility, penetration-enhancing properties, and ability to solubilize both hydrophilic and hydrophobic drugs, *DMSO* is widely used in ophthalmic applications [40–42]. Its inclusion in corneal preservation solutions further demonstrates its biocompatibility and relatively lower toxicity compared to other organic solvents [41, 43], making it a promising choice for *ISFSI* systems.

Furthermore, a major challenge associated with controlled-release formulations is the *burst-release* effect, where rapid drug diffusion within 24 h, particularly before polymer solidification, leads to potential toxicity, suboptimal longterm release, and reduced formulation efficiency [31, 44]. This effect is more pronounced in *ISFSI* due to highly watermiscible solvents accelerating drug diffusion into surrounding tissues [44]. To mitigate this, solid lipids, liquid lipids, surfactants, and polymer cross-linking agents are incorporated to regulate drug release [33, 45–48]. Solid lipids form hydrophobic matrices that reduce implant porosity and slow aqueous fluid penetration [49], liquid lipids enhance drug solubility while modulating *burst release*, while surfactants and polymer cross-linking agents further refine implant microstructure, reducing drug diffusion rates [31, 48]. Optimizing these strategies minimizes *burst release*, improving drug retention and therapeutic efficacy [44]. Therefore, various drug release retarder candidates were incorporated into the formulations in this study to address the initial burst release, ensuring a steady and prolonged drug release profile.

This study aimed to develop and characterize a *ketotifen fumarate*-loaded *PLGA*-based *ISFSI* for sustained ocular drug delivery for both human and veterinary animal species. The proposed system is designed to provide controlled and prolonged drug release, improved ocular bioavailability compared to conventional eye drops, enhanced patient compliance by minimizing dosing frequency, and reduced systemic exposure and potential side effects. The feasibility of this novel delivery system was assessed through physicochemical characterization, *in vitro* drug release studies, and biocompatibility assessments.

## Materials and Methods

### Materials

*Ketotifen hydrogen fumarate* (*Letco*^™^ lot #2,103,160,085) was procured from Letco Medical, LLC (Decatur, AL, USA). *Poly (D, L-lactide-co-glycolide) (PLGA)* which has a 50:50 lactide-to-glycolide ratio and is end-capped with ester terminal (*Resomer*^®^ RG 503; Mw 24,000–38,000), was purchased from Sigma-Aldrich (St. Louis, MO, USA). *Kolliphor*^®^ *RH 40* was kindly provided by BASF (Florham Park, NJ, USA). *Labrasol*^®^ *(caprylocaproyl macrogol-8 glycerides EP)* and *Maisine*^®^ (*glyceryl monolinoleate*) were kindly donated by Gattefosse (Lyon, France).

Whatman^®^ nylon syringe filters (0.2 μm and 0.45 nm), phosphate-buffered saline (PBS tablet, 100 mL pH 7.4; Lot #20E0456086), *dimethyl sulfoxide (DMSO,* ACS grade), and *acetonitrile* (HPLC grade) were sourced from VWR International (Suwannee, GA, USA). Milli-Q water was generated using an in-house Millipore water purification system (St. Louis, MO).

The human corneal epithelial cell line (HCE-T) was obtained from ATCC (Manassas VA), and these subcultures were generously provided by the College of Veterinary Medicine, Auburn University. *Alamar Blue*^*®*^ was purchased from Bio-Rad Antibodies (Raleigh, NC, USA), and *Calcein AM* (*Calcein acetoxymethyl ester*) and *Propidium Iodide* (PI) were obtained from BD Biosciences (San Jose, CA, USA). Dulbecco's Modified Eagle Medium/Nutrient Mixture F-12 (DME/F-12), fetal bovine serum (FBS, 10%), and penicillin–streptomycin (1%) were supplied from Thermo Scientific (Waltham, MA, USA).

## Methods

### Preparation of *Ketotifen Fumarate*-loaded ISFSIs

*Ketotifen fumarate*-loaded *PLGA*-based *ISFSI* formulations were prepared using the solvent-induced phase inversion method [29]. Each formulation contained 5% w/w *ketotifen fumarate*, as outlined in Table I. *PLGA* with a 50:50 *lactide-to-glycolide* ratio was selected as the polymer, and *DMSO* was used as the solvent for both the polymer and drug. The concentration of 30% PLGA was used for the ISFSIs based on our preliminary study in comparing initial burst release at 24 h for different PLGA conditions and release-modifying agents as shown in Supplementary Figure S1.

To modulate drug release, three different lipid-based excipients as release retarders, selected based on their miscibility with DMSO as the polymer solvent, i.e., *Maisine*^®^, *Labrasol*^®^, and *Kolliphor*^®^ RH 40, were incorporated. First, a 30% w/w *PLGA* solution was prepared in DMSO or DMSO-liquid lipid mixtures by vortex mixing for 1 min and sonication for 1 h. *Ketotifen fumarate* was then accurately added to reach a final concentration of 5% w/w, followed by 30 min of sonication.

### High-Performance Liquid Chromatography (HPLC) Analysis

#### Chromatographic Conditions

*Ketotifen fumarate* quantification was performed using a Waters HPLC system (Alliance e2695) with an autosampler, 2998 PDA detector, and a column compartment set at a controlled temperature. The separation was carried out on a Phenomenex Luna^®^ 5 μm C18(2) particles with 100 Å pore size LC column (150 × 4.6 mm, P/No: 00 F-4252-E0). The mobile phase, composed of methanol and water (70:30 v/v) with 0.035% *triethylamine* (TEA), was run at a flow rate of 1 mL/min. A 20 μL sample was injected, with the system run time set to 10 min and detection at 300 nm wavelength. The column temperature was maintained at 30 °C, and isocratic elution was employed for the separation [50]. Data processing was done using Empower^®^ 3 software (Waters Corp., Milford, MA, USA).

System suitability was assessed by injecting a standard solution of *ketotifen fumarate* (100 μg/mL) into the HPLC system in six replicates. The evaluation included the tailing factor, theoretical plate number, and peak area reproducibility. The tailing factor was required to be ≤ 2.0 to ensure a symmetric peak shape. Column efficiency was assessed by calculating the number of theoretical plates (USP method), with acceptable values set at ≥ 2000. The relative standard deviation (%RSD) of the peak areas from the replicate injections was calculated to assess precision, with a target RSD of ≤ 2.0%. These parameters confirmed that the chromatographic system was suitable for routine analysis of *ketotifen fumarate* in the ISFSI samples.

A standard calibration curve for *ketotifen fumarate* was generated using solutions with concentrations ranging from 0.5 to 200 μg/mL, with each concentration analyzed in triplicate. The regression analysis was conducted via the least-squares method, and a correlation coefficient (R^2^) of ≥ 0.999 was considered satisfactory. The limit of detection (LOD) and limit of quantification (LOQ) were determined by applying the residual standard deviation of the regression data derived from the HPLC calibration curve. The criteria of LOD = 3.3 × (RSD/b) and LOQ = 10 × (RSD/b), where RSD refers to the residual standard deviation and b is the slope of the regression line. These calculations assume that the measured concentrations follow a normal distribution [51]. Precision was determined by injecting six replicate samples of the *ketotifen fumarate*-loaded ISFI formulation, each containing 100 μg/mL of the drug. The percentage recovery and RSD were calculated to assess repeatability. The low RSD value and recovery close to 100% indicated excellent precision and reproducibility of the method.

### Evaluation of *Ketotifen Fumarate*-loaded ISFSIs

#### Visual Appearance

The *ISFSI* formulations were visually examined at room temperature against a white background. They were expected to appear clear, transparent to yellowish, and free of any particulate matter or precipitation.

#### Viscosity

Viscosity measurements were performed using Brookfield DV-II + Pro Viscometer (Brookfield Engineering Labs Inc, Middleboro, MA, USA). Briefly, 0.5 mL of each *ISFSI* formulation was placed in the cone-and-cup plate chamber, using spindle 40z at 25 °C. Viscosity (in Pa·s) was recorded at 20 rpm, with triplicate measurements.

#### Solidifying Time

To evaluate solidification time, 200 μL of each formulation was injected into 20 mL PBS (pH 7.4) in a glass vial, which was placed in a thermostatically controlled water bath (Unimax, IKA, Germany) at 37 °C. The solidification time was defined as the time needed for the formulation to solidify completely within the PBS solution. Each formulation was tested in triplicate.

#### Assay (Percentage of Recovery) of Ketotifen Fumarate in ISFSI

For *ketotifen fumarate* recovery, 20 mg of each formulation was dissolved in 10 mL acetonitrile. After sonication for 30 min, the solution was filtered using a 0.2 μm nylon syringe filter and then analyzed by HPLC. The percentage of recovery was calculated as:

$$\%\;\mathrm{Drug}\;\mathrm{Recovery}\;=\frac{\mathrm{Amount}\;\mathrm{of}\;\mathrm{drug}\;\mathrm{recovered}}{\mathrm{Initial}\;\mathrm{amount}\;\mathrm{of}\;\mathrm{drug}}\times 100$$

Each formulation was tested in triplicate.

### Drug Release of *Ketotifen Fumarate*-loaded *ISFSIs*

#### *In Vitro* Drug Release Study

The *in vitro* release of *ketotifen fumarate* from the *ISFSIs* was carried out using the ‘Sample and Separate’ method [52]. Precisely 50 μL of each formulation was deposited at the bottom of a centrifuge tube, followed by the gentle addition of 5 mL of phosphate-buffered saline (PBS, pH 7.4). The tubes were then incubated in a static incubator (Unimax, IKA, Germany) at 37 ± 0.5 °C to simulate physiological conditions.

At predetermined time points (1, 3, 5, and 7 days, then weekly up to 10 weeks), the entire 5 mL release medium was carefully withdrawn and replaced with an equal volume of fresh PBS to maintain sink conditions. The retrieved samples were subsequently examined using a validated HPLC method at 300 nm to quantify the released *ketotifen fumarate*. All experiments were conducted in triplicate.

#### Analysis of the Initial Burst Release and Drug Release Kinetics

The initial burst release was measured as the cumulative drug release at 24 h, representing the rapid diffusion phase. Extended-release kinetics were analyzed using five models: zero-order, first-order, Higuchi, Hixson-Crowell, and Korsmeyer-Peppas. Zero-order kinetics were assessed by plotting cumulative release over time, while first-order kinetics were evaluated by graphing the logarithm of the remaining drug concentration. The Higuchi model was used to describe diffusion-driven release, with data plotted against the square root of time. The Hixson-Crowell model examined dissolution rate changes by plotting the cube root of the remaining drug amount over time. The Korsmeyer-Peppas model, which differentiates between Fickian and non-Fickian diffusion, was applied by plotting log cumulative release versus log time.

Curve fitting and statistical analyses were performed using Microsoft Excel 365, and the best-fitting model for each formulation was determined based on regression coefficients (R^2^) and goodness-of-fit evaluation. The kinetic parameters obtained provided insights into the drug release behavior and mechanisms governing *ketotifen fumarate* release from the *ISFSIs*.

#### pH Changes of the Release Medium During the Release Study

The pH of the release medium was monitored throughout the drug release study to assess potential changes due to polymer degradation and drug release. At predefined time points (1 h, then 1, 3, 5, 7, 14, 21, and 28 days), the pH of the collected medium was measured using a calibrated pH meter (Accumet^®^ XL150 pH/mV, Fisher Scientific, USA) at room temperature (~ 25 °C). Before each measurement, the pH meter was calibrated using standard buffer solutions (pH 4.0, 7.0, and 10.0). Each experiment was performed in triplicate.

#### Wet Weight of the Solidified ISFIs

The wet weight of the solidified *ISFSIs* was evaluated over time by immersing the implants in phosphate-buffered saline (PBS, pH 7.4) at 37 °C, similar to the *in vitro* release study. At predefined time points (1 h, 1, 3, 5, 7, 14, 21, and 28 days), the entire release medium was removed, and the solidified implants were carefully retrieved from PBS. They were gently blotted with filter paper to remove excess surface moisture and immediately weighed. The solidified implants were then re-immersed in fresh PBS and incubated at 37 °C. Each experiment was conducted in triplicate.

### Characterization of the Selected Formulation of Ketotifen Fumarate-loaded ISFSI

#### Differential Scanning Calorimetry (DSC)

Thermal analysis of *ketotifen fumarate*, *PLGA*, physical mixture of *PLGA*-*ketotifen fumarate* (1:1), *Maisine*^®^, liquid *ISFSI*, and solidified *ISFSI* was performed using DSC Q200 Calorimeter (V24.10, TA Instruments, New Castle, DE). Samples (2–5 mg) were sealed in non-hermetic aluminum pans and analyzed under nitrogen from 25 °C to 300 °C at a 10 °C/min heating rate. An empty aluminum, also sealed with a lid, served as the reference.

#### Scanning Electron Microscopy (SEM)

The surface morphology of the solidified ISFSI was analyzed using SEM (Zeiss EM10, Hebron, KY). The solidified implant was left to air dry overnight on filter paper. The dried sample was then mounted onto metal stubs using double-sided carbon tape. A thin layer of gold was applied to the samples before imaging under SEM at an excitation voltage of 20 kV.

#### Rheological Behavior

The rheological behavior of the selected *ISFSI* formulation was determined using a Bohlin CVO rheometer (Worcestershire, UK). A sample of 6 mL from the selected formulation was placed into the cup and bob chamber of the rheometer using a concentric cylinder system (C14 DIN 53019). The study was conducted at the shear rate ranging from 0 to 1500 s^−1^. The temperature was set at 25 °C. The flow behavior of the examined formulations was determined by plotting the logarithm of shear stress against the logarithm of shear rate, based on the power law model:

LogS=Logk+nLogD

where D represents the shear rate (s^−1^), S is the shear stress (Pa), n denotes the power law constant, and k corresponds to the consistency index (Pa·s). The value of n, obtained as the slope from the log S *vs.* log D plot, characterizes the flow type. A value of *n* = 1 indicates Newtonian flow, whereas *n* < 1 suggests shear-thinning behavior. Conversely, if *n* > 1, the formulation exhibits a shear-thickening flow [53].

#### Injectability Parameters Prediction

Injectability parameters such as flow rate and injection force were predicted using a mathematical approach of the power law, using the sample’s rheological data [54–56]. The following equation was used to estimate the injection force for power-law fluids:

$$F_{v}≅2{\left(\frac{3n\;+\;1}{n}\right)}^{n}\pi^{1-n}k\;l_{n}Q^{n}\frac{R_{p}^{2}}{R_{n}^{3n+1}}$$

In this context, *F*v denotes the viscous contribution to the injection force (Newton or N), n and k refer to the power law constant and consistency index determined from the rheological data using power law model, *l*n is the length of the needle (mm), *Q* represents the volumetric flow rate (mm^3^/s), *R*p is the radius of the syringe barrel (mm), and *R*n is the inner radius of the needle (mm). The *Q* value can be calculated using the equation below:

$$Q=\frac{n\dot{\gamma }\pi R_{n}^{3}}{3n\;+\;1}$$

In this equation, $\dot{\gamma }$ represents the shear rate (s^−1^). The calculations were performed using a 1 mL syringe, with a barrel radius (*R*p) ranging from 0.18 to 0.19 inches [57, 58]. The needle dimensions listed in Supplementary Table S1 were used to estimate the flow rates and injection forces. A plot of injection force (N) *versus* shear rate (s^−1^) was generated for each combination of syringe and needle dimensions. The maximum permissible injection force is 40 N [59].

### *In Vitro* Cytotoxicity Study of Ketotifen Fumarate-loaded ISFI

The cytotoxicity of the leachable drug extract from the ISFSI was evaluated using the Alamar Blue assay and Calcein AM/Propidium Iodide (PI) staining in *human corneal epithelial cells (HCE-T).*

The leachable drug extract was obtained by incubating the solidified ISFSIs in phosphate-buffered saline (PBS, pH 7.4) at 37 °C, like the *in vitro* release method, where at the predetermined time points (1, 3, and 5 days, then weekly up to 5 weeks), the liquid-containing drug extracts were collected by collecting the entire release medium (5 mL) and replaced with fresh medium. All the collected samples were filtered using a 0.2 μm syringe filter and stored at 4 °C until use.

### Assessment of Cell Viability Via Alamar Blue Assay

HCE-T cells were maintained in a DME/F-12 medium supplemented with 10% fetal bovine serum (FBS) and 1% penicillin–streptomycin at 37 °C in a humidified incubator with 5% CO_2_. Cells were seeded in 96-well plates at a density of 5 × 10^3^ cells per well and allowed to attach and stabilize overnight in a controlled environment. Cell viability was assessed using the Alamar Blue assay (resazurin reduction method). The experimental samples, consisting of the prepared leachable drug extracts and PBS pH 7.4 at different dilutions (4 × and 10 ×), were added to the cells and incubated for a 48-h period. Untreated cells (culture medium only) served as the control. Following this incubation, 5 μM of Alamar Blue was added directly to each well, and the plates were further incubated for 4 h to allow for sufficient metabolic conversion of the dye by viable cells.

The assay's optical readings were taken using a fluorescence microplate reader, with an excitation wavelength set at 540 nm and an emission wavelength at 590 nm. To quantify cell viability, the fluorescence intensity values from the treated cells were compared to those from the untreated control cells. The percentage of cell viability was calculated using the following formula:

$$\mathrm{Cell}\;\mathrm{viability}\;(\%)=\frac{\mathrm{Fluorescence}\;\mathrm{intensity}\;\mathrm{of}\;\mathrm{the}\;\mathrm{treated}\;\mathrm{cells}}{\mathrm{Fluorescence}\;\mathrm{intensity}\;\mathrm{of}\;\mathrm{the}\;\mathrm{control}}\times 100$$

### Assessment of Cell Viability and Death Via Calcein-AM/PI Staining

To further analyze the cytotoxicity effects, Calcein-AM/PI staining was performed 24 h post-treatment. Each well of the 96-well plate contained 1 × 10^4^ cells to ensure adequate detection sensitivity. The staining protocol was initiated by applying a solution containing Calcein-AM and PI to distinguish between live and dead cells. Viable cells were identified by their green fluorescence, emanating from the esterase activity within live cells that converts Calcein-AM into a green fluorescence product. Conversely, dead cells were marked by red fluorescence, indicative of PI binding to DNA within cells compromised by membrane integrity loss. After staining, the cells were rinsed with PBS to eliminate any residual dye and minimize background fluorescence. The images were then captured using a confocal microscope (Nikon) equipped with appropriate filters for green and red fluorescence.

### Statistical Analysis

All experiments were conducted in triplicate (*n* = 3), and data were presented as mean ± standard deviation (SD). Statistical analyses were performed using Microsoft Excel 365 and BioRender Graph. One-way ANOVA with Tukey’s multiple comparison test was used for group comparisons. A p-value of < 0.05 was considered statistically significant.

## Results

### Linearity and Sensitivity Determination of the HPLC Method

The HPLC method used in this study successfully generated a well-separated and distinct peak for *ketotifen fumarate* in the chromatogram, as shown in Supplementary Figure S2, ensuring reliable detection and quantification. To validate the analytical method, linearity, LOD, and LOQ were determined. The linearity of *ketotifen fumarate* was assessed across a broad concentration range of 0.5–200 μg/mL, with the calibration curve exhibiting an excellent coefficient of determination (R^2^ = 0.9999), as presented in Supplementary Figure S3. This strong linear relationship confirms the accuracy and reliability of the method for quantifying *ketotifen fumarate* over the tested concentration range. Chromatographic performance was also satisfactory, with an acceptable tailing factor and theoretical plate count (USP), reflecting good system suitability. As summarized in Table II, the validated HPLC–UV method exhibited high sensitivity, with the calculated LOD and LOQ determined to be 0.81 μg/mL and 2.45 μg/mL, respectively. These values confirm the method’s capability to detect and quantify low concentrations of *ketotifen fumarate* in the samples. Precision was evaluated through six replicate injections of the *ketotifen fumarate*-loaded ISFI formulation (equivalent to 100 μg/mL of *ketotifen fumarate*), yielding a mean recovery of 100.43 ± 0.53% and an RSD of 0.53%. These results validate the HPLC method as a precise, sensitive, and robust analytical tool for quantifying *ketotifen fumarate* in the developed ISFSI formulations.

### Physicochemical Evaluations of ISFSI Formulations

**Fig. 1:**
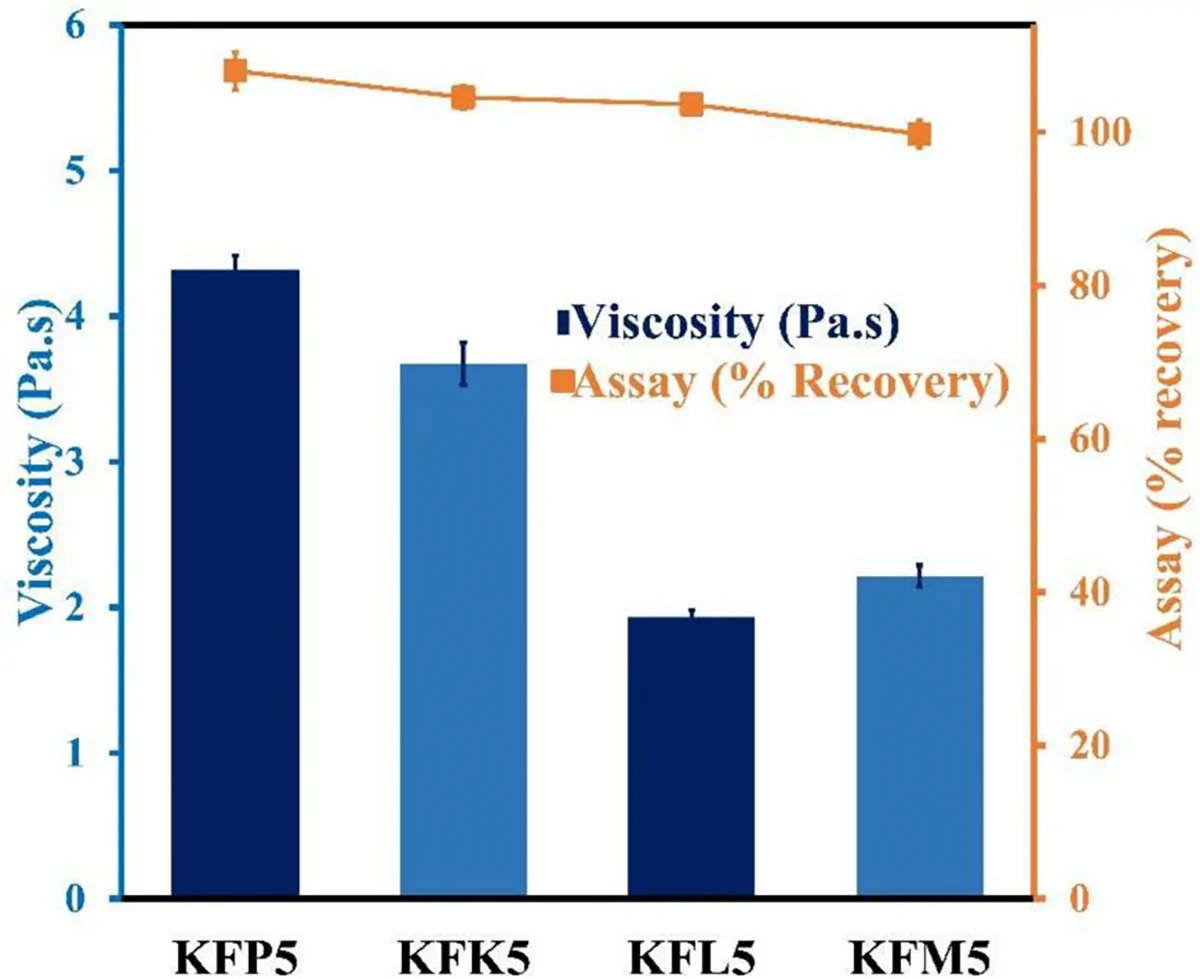
Drug recovery (%) and viscosity at 20 rpm of the prepared *ISFSIs*

All prepared *ketotifen fumarate*-loaded ISFSI formulations—KFP5, KFK5, KFL5, and KFM5—were clear, yellowish liquids at room temperature. Upon injection into an aqueous medium at 37 °C, they initially formed a gel, which then gradually solidified within less than one minute. This two-step transformation ensures that the formulation remains localized at the administration site, allowing for a stable implant formation that facilitates controlled and prolonged drug release. Figure 1 presents the drug assay and viscosity at 20 rpm for the prepared ISFSIs. The viscosity of the formulations varied depending on the formulation composition. KFP5 exhibited the highest viscosity (~ 4.5 Pa·s), followed by KFK5 (~ 3.8 Pa·s), while KFM5 and KFL5 showed lower viscosities (~ 2.5 and ~ 2.0 Pa·s, respectively). These differences might influence the drug-release character of the ISFSIs, also affecting injectability, gelation dynamics, and *in situ* solidification. Despite these variations in viscosity, all formulations maintained a drug content above 95%, demonstrating successful drug encapsulation with no significant degradation or loss during formulation.

### Initial Burst Release and Cumulative Drug Release Profiles

**Fig. 2:**
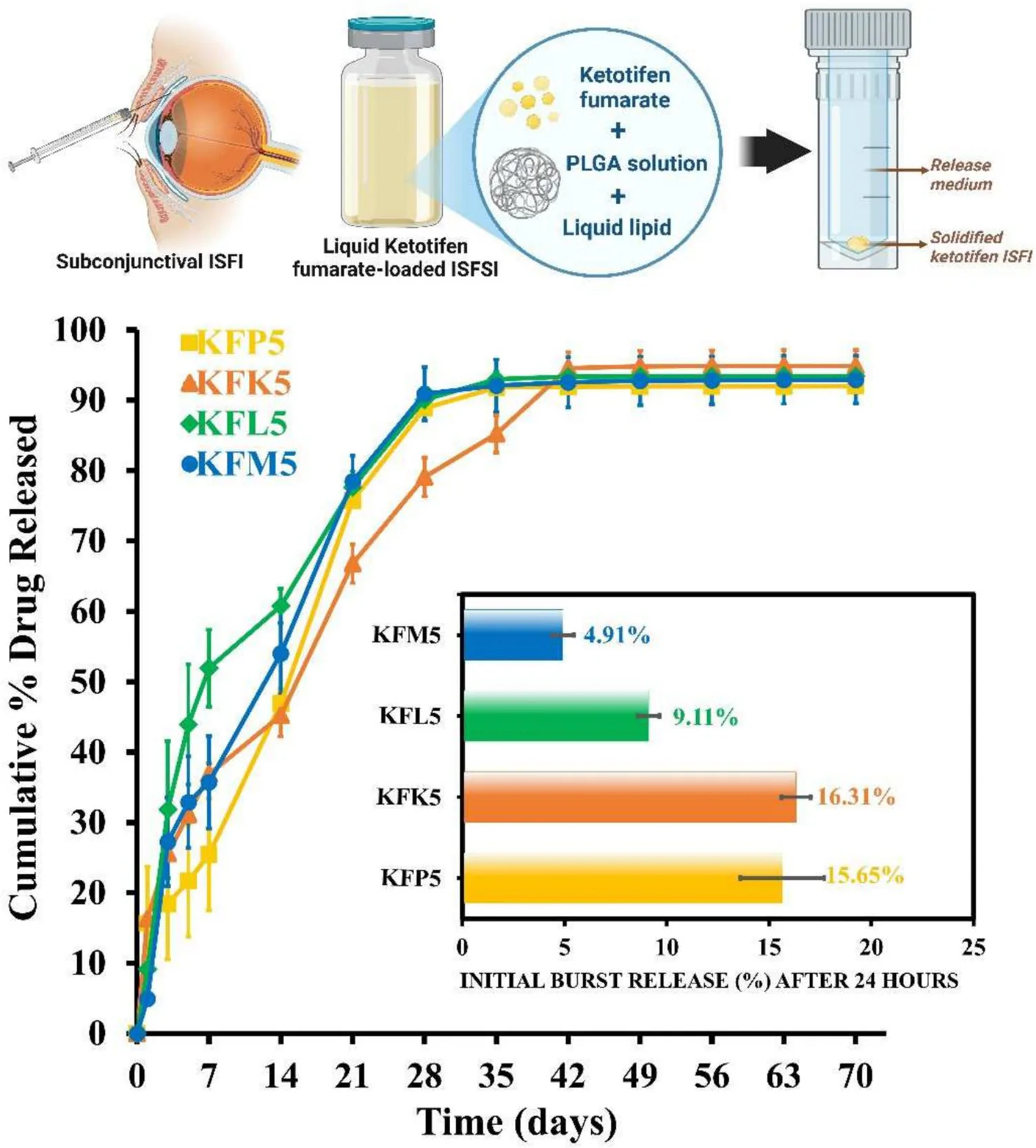
Initial burst release at 24 h and cumulative drug release over 10 weeks for 5% *ketotifen fumarate*-loaded ISFSIs: KFP5 (without a release-modifying agent), KFK5 (with *Kolliphor*^®^ RH 40), KFL5 (with *Labrasol*^®^), and KFM5 (with *Maisine*^®^)

The initial burst release at 24 h and the cumulative drug release profiles over 10 weeks were analyzed to assess the effect of drug release retarders and excipient interactions on the drug release behavior of each ISFSI formulation. As shown in Fig. 2, more than 80% of the drug was released within 35 days (5 weeks), with variations observed among the formulations. Over the 10-week study period, KFK5 and KFL5 exhibited faster cumulative drug release (~ 94.88% and ~ 93.41%), whereas KFP5 (91.98%) and KFM5 (92.89%) demonstrated a more sustained release profile.

The formulations followed a triphasic drug release pattern, characterized by an initial rapid release within the first week, a slower release phase by the third week, and a second rapid release phase by the fifth week. The control formulation (KFP5) showed a higher burst release (15.65%), while KFK5 displayed the highest burst release (16.31%) among all formulations. In contrast, KFL5 and KFM5 exhibited reduced burst release, with 9.11% and 4.91%, respectively.

The cumulative drug release profiles further differentiated the formulations based on their release rates. KFP5 exhibited a moderately rapid drug release in the first week, with approximately 25% of the total drug released within this period. Between days 7 and 28, the drug release followed a steady, diffusion-controlled phase, after which a slight acceleration was observed beyond day 28. KFK5 followed a similar triphasic release pattern, with ~ 37% drug release within the first 7 days. The release rate remained steady between days 7 and 28, followed by an increase in drug release beyond day 28. KFL5 showed a rapid initial drug release, with ~ 53% released within the first 7 days. The second phase, from days 7 to 28, followed a steady diffusion-controlled release. After day 28, drug release showed a gradual increase, though less pronounced than in KFK5. KFM5 demonstrated moderate initial drug release, with ~ 35% released in the first 7 days. Between days 7 and 28, drug release remained stable and diffusion-controlled. After day 28, a mild increase in drug release was observed, though less significant compared to the other formulations.

### Drug Release Kinetics

**Fig. 3:**
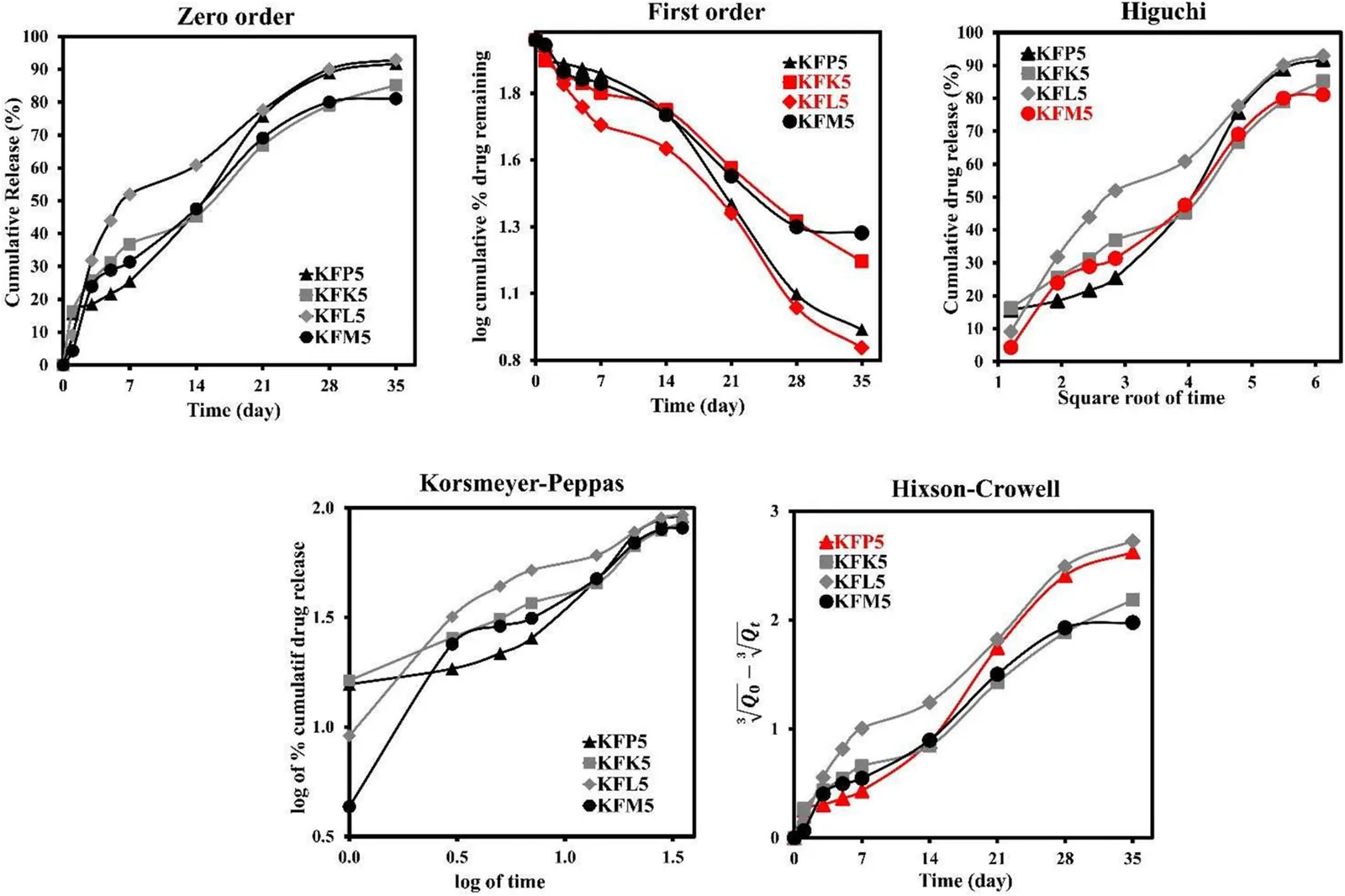
*In vitro* release kinetics of KFP5, KFK5, KFL5, and KFM5 fitted to various mathematical models: Zero-order, First-order, Higuchi, Korsmeyer-Peppas, and Hixson-Crowell. The red color indicates the best-fitting model

The *in vitro* drug release kinetics of KFP5, KFK5, KFL5, and KFM5 were evaluated using mathematical models, including Zero-order, First-order, Higuchi, Hixson-Crowell, and Korsmeyer-Peppas models (Fig. 3, Table III). The bestfit model for each formulation was determined based on the highest coefficient of determination (R^2^) values.

The First-order model provided the best fit for KFK5 (R^2^ = 0.9832) and KFL5 (R^2^ = 0.9822), indicating that drug release from these formulations was concentration-dependent, with the rate decreasing as drug concentration declined over time. The Hixson-Crowell model exhibited the highest R^2^ value for KFP5 (0.9792), suggesting that its release was influenced by polymer erosion and a gradual reduction in implant surface area over time. The Higuchi model demonstrated a strong correlation with KFM5 (R^2^ = 0.9798), indicating that drug release was primarily diffusion-controlled, following a square root of time-dependent release profile. The Korsmeyer-Peppas model was applied to further evaluate the drug release mechanism, particularly the contribution of diffusion and polymer relaxation. The release exponent (n) values ranged from 0.47 to 0.76, indicating non-Fickian (anomalous) transport, where both diffusion and polymer relaxation contributed to drug release. KFM5 exhibited the highest n value (0.76), suggesting a more diffusion-driven release mechanism, while KFK5 had the lowest n value (0.47), indicating a stronger influence of polymer relaxation. These results indicate that KFP5 follows an erosion-driven release profile, KFK5 and KFL5 exhibit concentration-dependent diffusion, and KFM5 demonstrates a diffusion-controlled release pattern with some contribution from polymer relaxation.

### pH Variations of the Medium During Drug Release

**Fig. 4:**
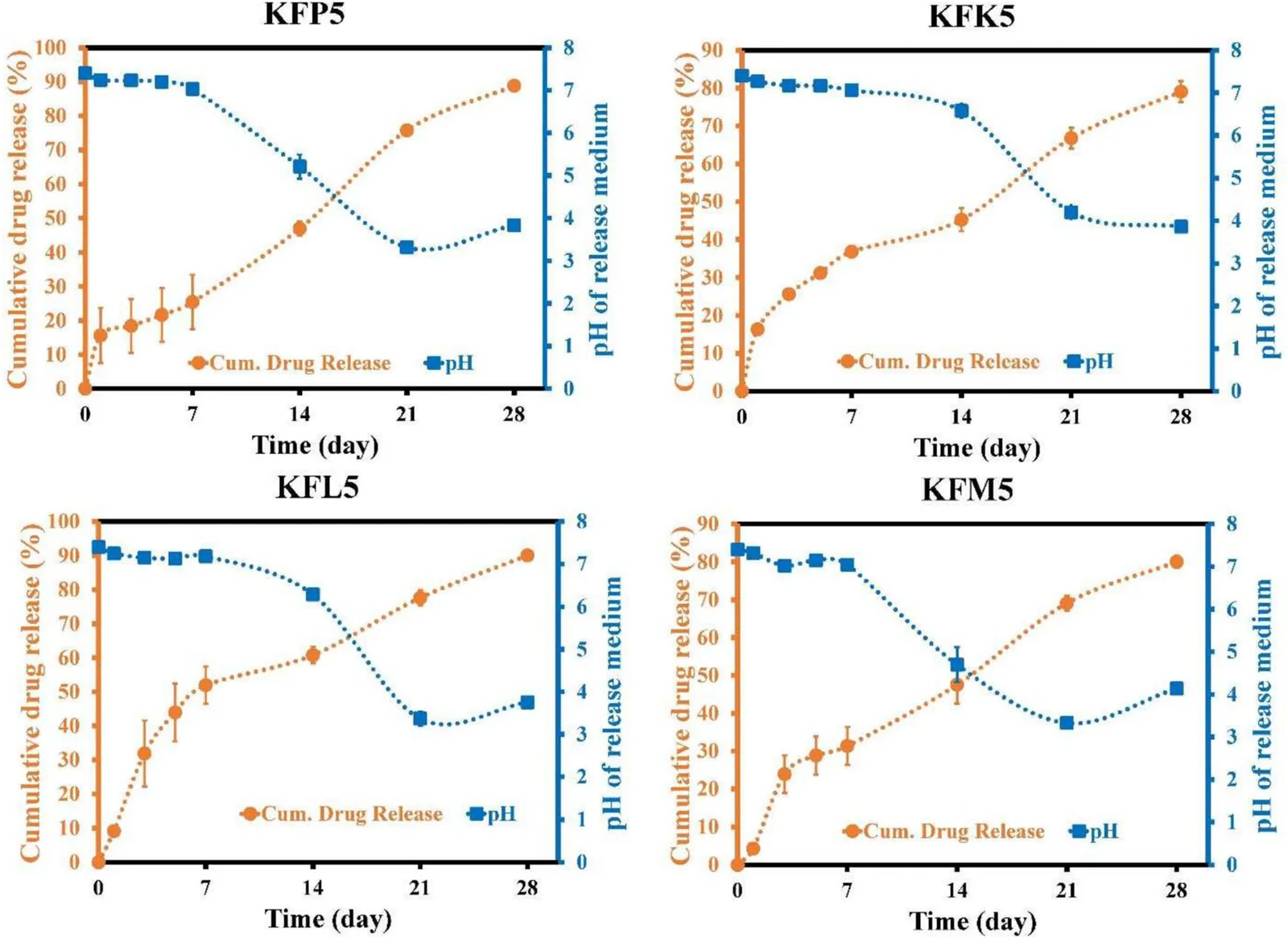
*pH* variations of the drug release medium in relation to the cumulative drug release over time at 37 °C for 4 weeks

The pH of the release medium was monitored over 4 weeks to evaluate whether polymer degradation led to acidic byproduct accumulation in relation to the cumulative drug release, as exhibited in Fig. 4. The results showed that all formulations exhibited a gradual pH decline, which is consistent with PLGA degradation and the formation of lactic and glycolic acids. KFP5 exhibited the most rapid pH drop (~ 3.5 by week 4), indicating accelerated polymer degradation, while KFM5 maintained a more stable pH (~ 5.5 by week 4), suggesting reduced acid accumulation.

### Wet Weight Changes and Polymer Degradation

**Fig. 5:**
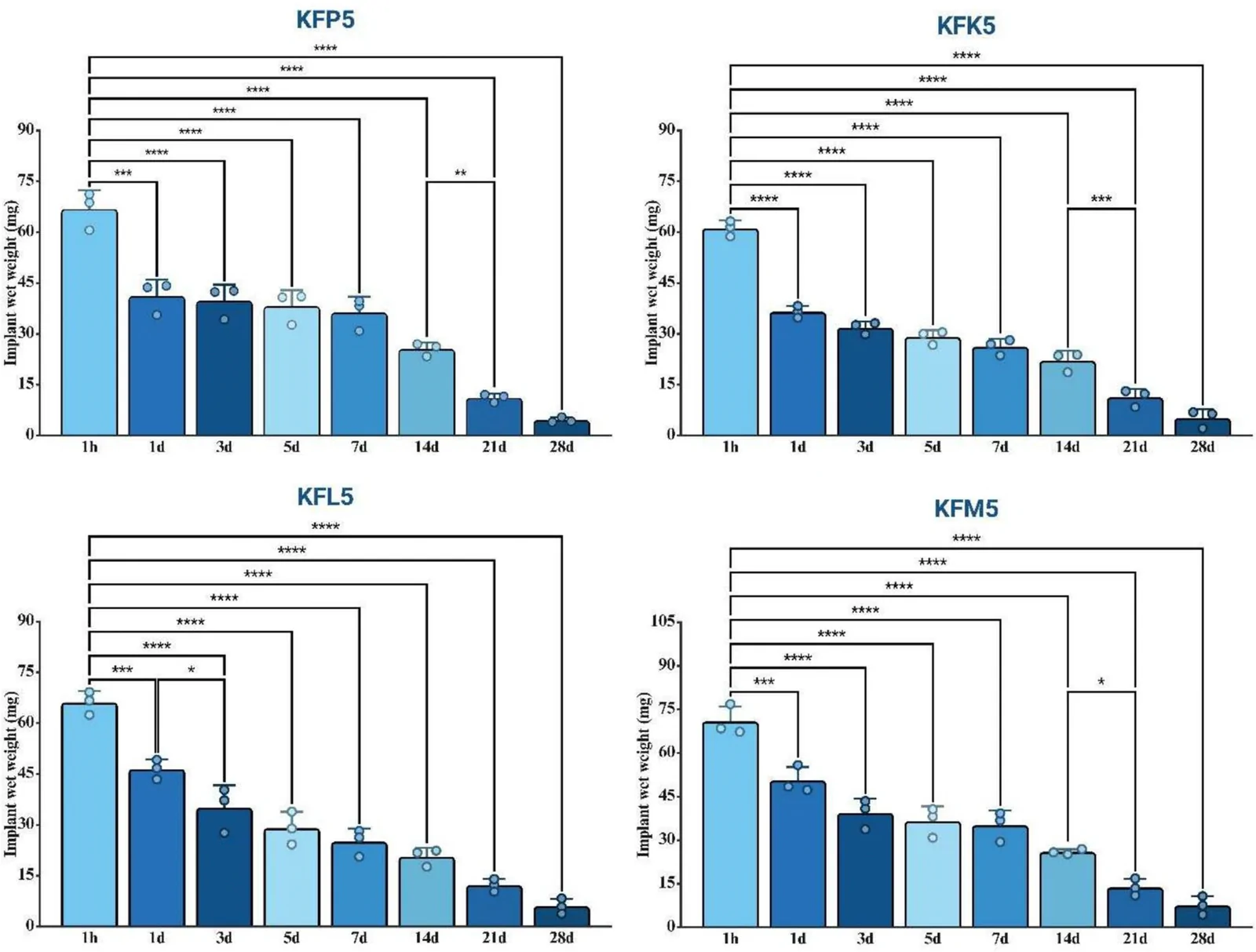
Wet weight changes of solidified ISFSIs overtime at 37 °C

The polymer degradation profile was further investigated by monitoring wet weight changes over 4 weeks, as described in Fig. 5. The results indicated that all formulations initially absorbed water within the first hour, corresponding to polymer swelling and hydration. Over time, a gradual reduction in wet weight was observed, indicative of polymer degradation. Among the formulations, KFP5 exhibited the most rapid weight loss, correlating with its higher burst release and polymer erosion-driven drug release. In contrast, KFM5 retained the highest wet weight over the study period, suggesting a slower degradation rate.

### Surface Morphological Features Using SEM

**Fig. 6:**
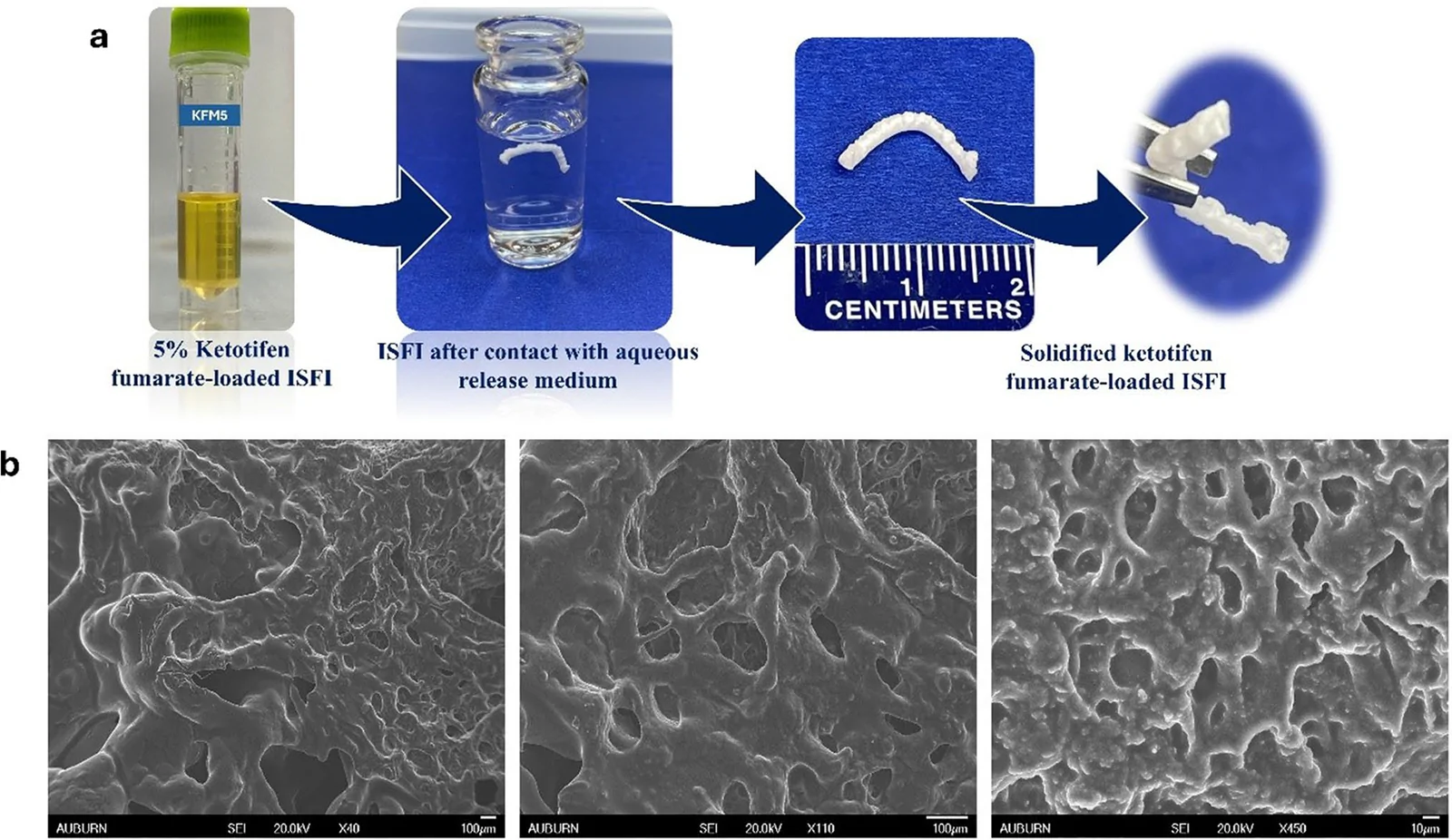
**a** Liquid and solidified *ketotifen fumarate*-loaded ISFSI; **b** Scanning Electron microscopy (SEM) micrographs of solidified *ketotifen fumarate*-loaded ISFSI at varying magnification levels: 10—450 times

To further investigate the structure of KFM5 after solidification, SEM was performed to examine its surface morphology. As shown in Fig. 6a, Solidified KFM5 formed a sponge and hollow structure. Furthermore, SEM micrographs in Fig. 6b of KFM5 displayed a highly porous microstructure, with an interconnected pore network that is critical for facilitating controlled drug diffusion. The formation of this porous network upon solidification suggests that polymer precipitation leads to a structured implant, allowing for sustained drug release while maintaining mechanical integrity.

### Rheological Behavior and Injectability Parameters

**Fig. 7:**
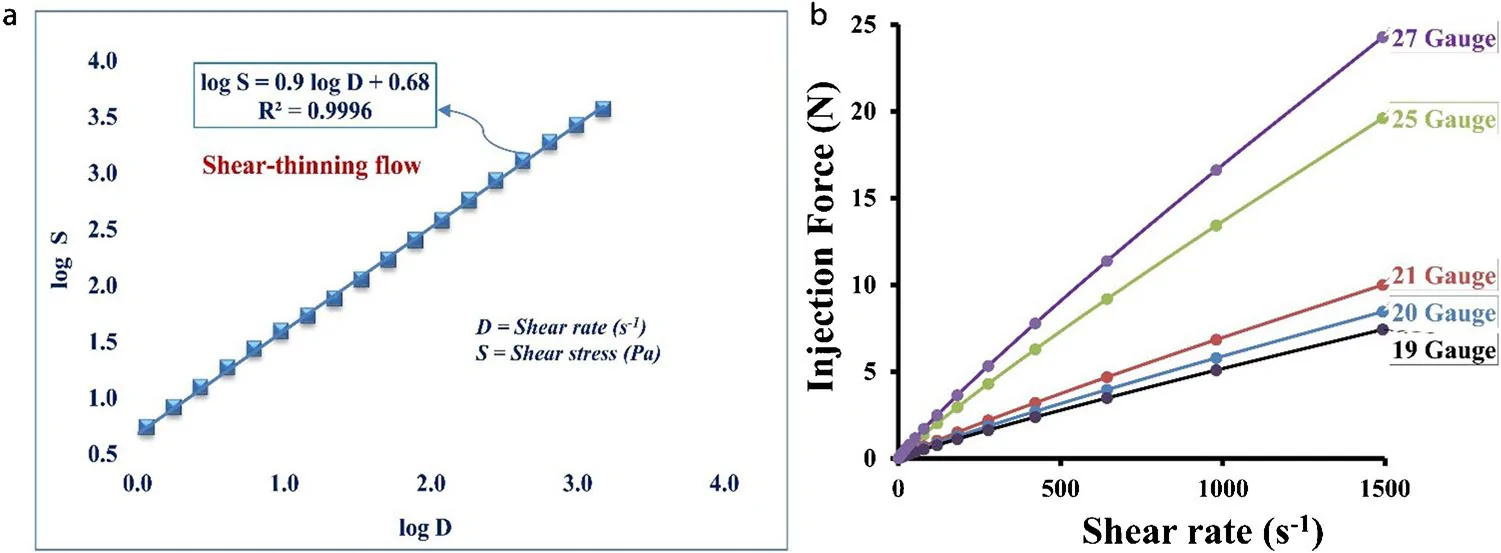
(**a**) Rheological behavior of liquid *ketotifen fumarate*-loaded ISFSI. (**b**) Predicted injection forces for *ketotifen fumarate*-loaded ISFSI using 5 different syringe and needle dimensions, calculated based on the power law model

The rheological behavior of KFM5 was evaluated using rheological analysis according to viscosity and shear of stress values of the sample in the rate of a shear range of 0–1500 s^−1^, where the formulation exhibited shear-thinning flow characteristics, with a linear relationship between logarithms of the shear stress and shear rate (R^2^ = 0.9996), power law index (*n*) value of 0.9 and consistency index of 4.8 Pa.s, as shown in Fig. 7a. The predicted injection force analysis in Fig. 7b and Table IV assessed the force required to administer KFM5 using different syringe and needle dimensions. The results demonstrated that 19G–25G needles provided the most favorable injection forces (< 20 N), ensuring ease of administration. The 27G needle required higher forces (24 N), though it still meets the clinically acceptable threshold of below 40 N.

### Thermal Analysis Using DSC

**Fig. 8:**
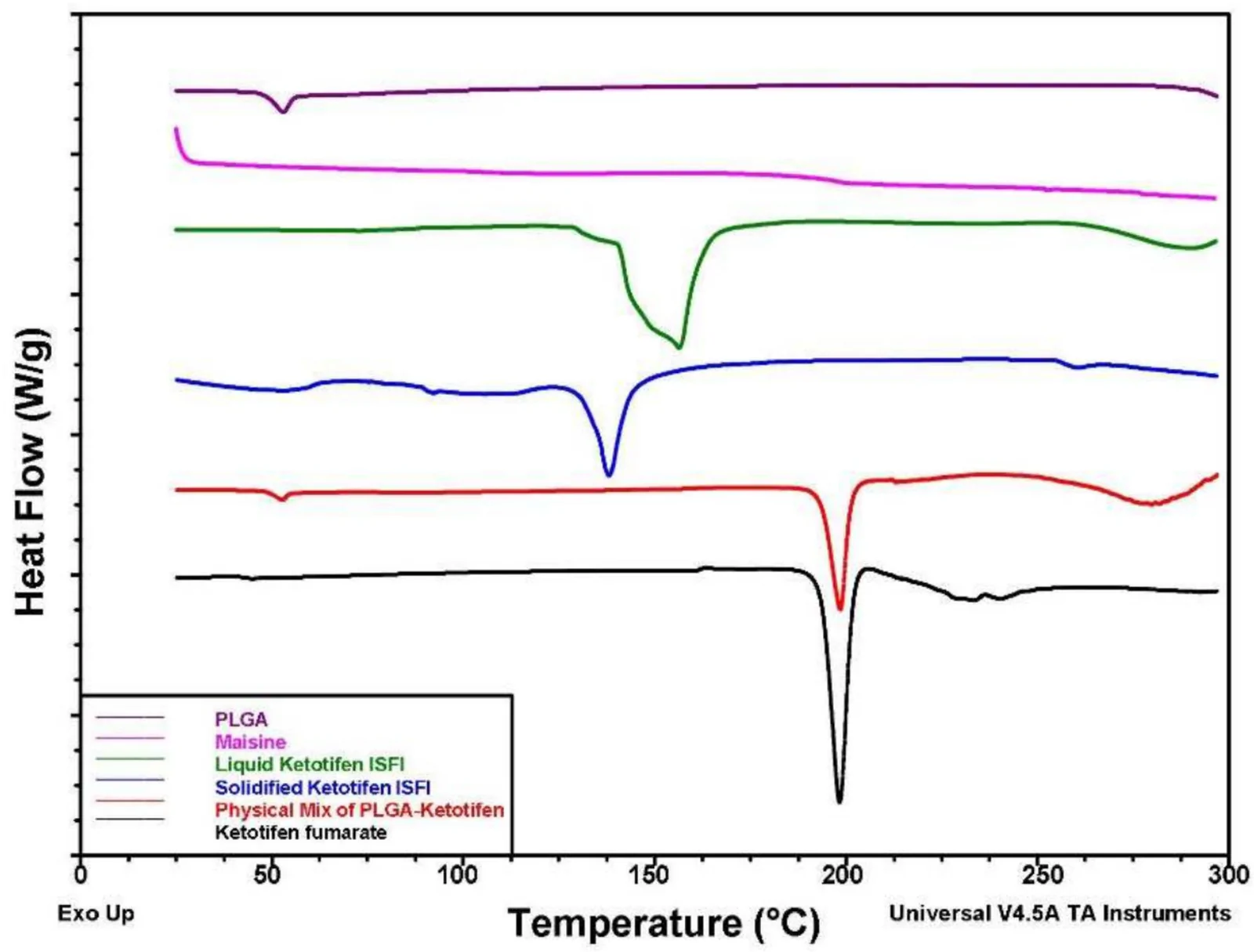
DSC thermograms of pure *ketotifen fumarate, PLGA, Maisine*^®^, the physical mixture of *PLGA-ketotifen fumarate*, and liquid and solidified KFM5

DSC analysis was conducted to evaluate the thermal behavior and physical state of *ketotifen fumarate* in KFM5. Figure 8 describes that pure *ketotifen fumarate* is crystalline with a single endothermic peak of a melting point of 198.28 °C, while pure PLGA and *Maisine*^*®*^ are amorphous. The physical mixture of *ketotifen fumarate* and PLGA did not alter the endothermic peak of *ketotifen fumarate*. The results confirmed that amorphization of *ketotifen fumarate* occurred on the liquid and solidified KFM5, as indicated by the absence of a sharp melting peak which is present in the pure crystalline drug. This transformation into an amorphous form enhances drug solubility and prevents crystallization-related instability in the formulation.

### *In Vitro* Biocompatibility Assessment

**Fig. 9:**
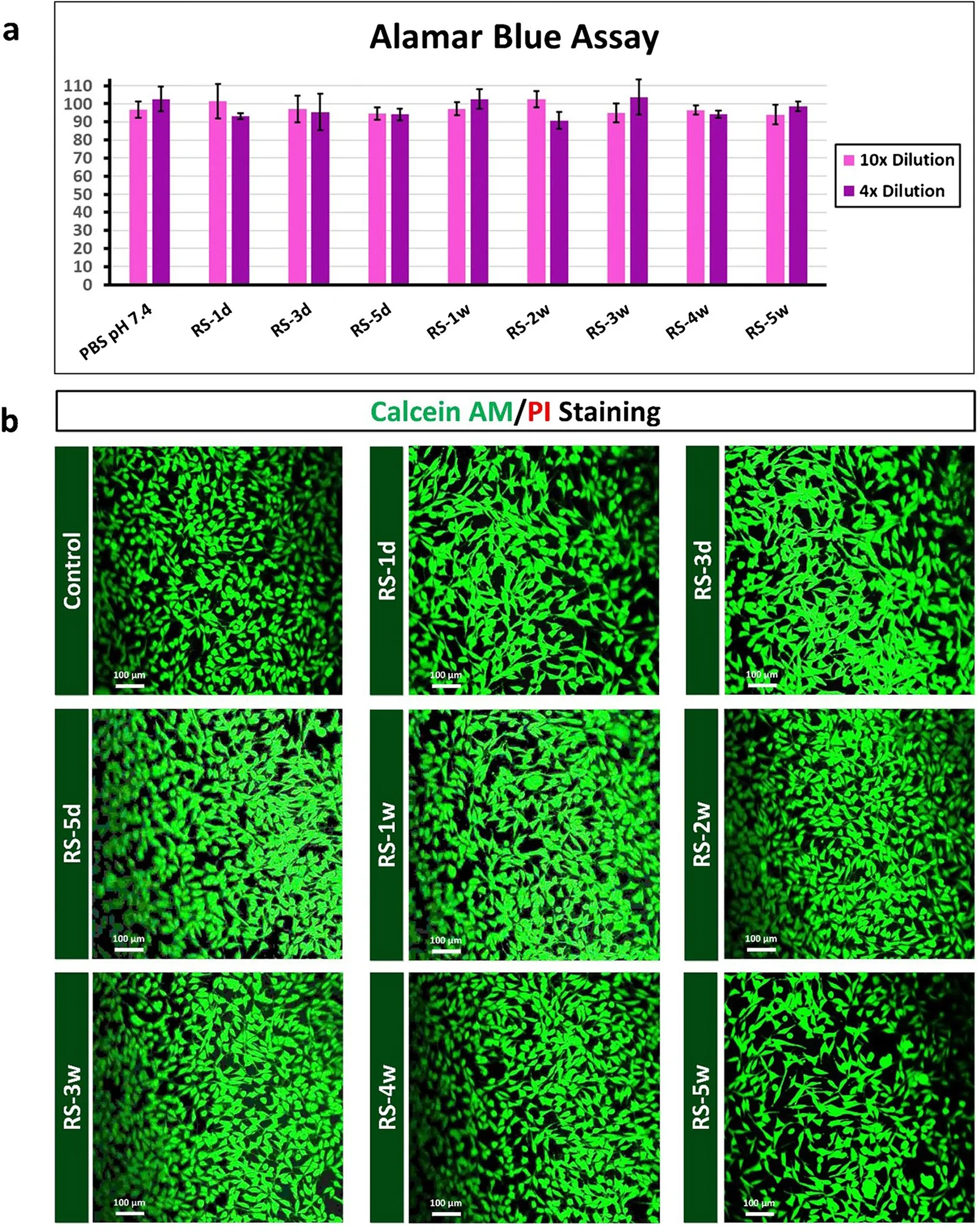
**a** Cell viability of *human corneal epithelial cells* (HCE-T) assessed using the Alamar Blue assay (Values represent mean ± standard deviation, *n* = 3). **b** Calcein AM/PI staining of *HCE-T cells* to determine the live and dead cells from the ISFSI formulation and control treatments

As shown in Fig. 9, the Alamar Blue assay revealed that cell viability remained above 90%, indicating that KFM5 does not exert significant cytotoxic effects on human corneal epithelial cells (HCE-T). Calcein AM/PI staining further confirmed a predominance of live cells (green fluorescence) with no detectable dead cells (red fluorescence), suggesting biocompatibility (no cytotoxic effects) with the cells.

## Discussion

The findings of this study highlight the potential of *ketotifen fumarate*-loaded *in situ* forming subconjunctival implants (ISFSIs) as a sustained ocular drug delivery system. Each formulation was assessed for its physicochemical evaluations, drug release characteristics, and biocompatibility to determine its suitability for long-term ocular administration. The results demonstrated that the choice of drug release retarder or other excipients and their interactions with the drug significantly influenced viscosity, drug release kinetics, polymer degradation, and cellular compatibility, ultimately impacting the formulation’s clinical potential.

The high drug recovery observed across all formulations confirms the efficiency of the preparation process, ensuring minimal drug loss and successful encapsulation of *ketotifen fumarate* within the polymer matrix. The viscosity differences among the formulations were observed, with KFP5 exhibiting the highest viscosity, while KFM5 and KFL5 displayed lower viscosities due to the inclusion of *Maisine*^*®*^ and *Labrasol*^*®*^. The lower viscosity in KFM5 suggests improved injectability, reducing the force required for administration, which was later confirmed in the injection force analysis. These findings are crucial, as an ISFSI formulation with lower viscosity enhances ease of administration and ensures uniform polymer deposition at the injection site, which is essential for effective drug release.

The initial burst release and cumulative drug release over 10 weeks provided insights into the impact of drug release retarders, as one of the excipients, on drug diffusion and polymer behavior. The control formulation, KFP5, exhibited a significantly higher burst release after 24 h compared to KFM5 and KFL5, indicating that the incorporation of *Maisine*^*®*^ and *Labrasol*^*®*^ successfully modulated the initial diffusion phase. A high burst release can lead to drug wastage and potential toxicity [44], particularly in ocular applications where sustained therapeutic levels are critical. The reduced burst release in KFM5 suggests that *Maisine*^*®*^ stabilized the polymeric network, thereby controlling the hydration process and minimizing premature drug diffusion.

Additionally, the cumulative drug release trends confirmed that KFM5 provided a more sustained and prolonged drug release compared to the control formulation, advancing the role of lipid-based excipients in controlling polymer erosion and drug diffusion. This result aligned with previous research [48] comparing *Labrasol*^*®*^ and *Maisine*^*®*^ in raloxifene HCl loaded *in situ* forming implant for bone injuries, where *Maisine*^*®*^ exhibited the most sustained and lowest initial burst release.

Drug release kinetics modeling further validated these observations by demonstrating that KFP5 followed the Hixson-Crowell model, indicating an erosion-driven release, while KFM5 aligned with the Higuchi model, confirming a diffusion-controlled mechanism. This distinction is important as erosion-based release may lead to unpredictable drug release rates due to variations in polymer degradation, whereas diffusion-controlled release ensures a steady and predictable drug release profile [60]. The SEM analysis of KFM5 provided additional evidence supporting the diffusion-based mechanism, as the micrographs revealed a highly porous structure with interconnected pores that facilitate sustained drug diffusion while maintaining implant integrity. The presence of this structured matrix upon solidification suggests that polymer precipitation during *in situ* gelation results in a scaffold-like implant, promoting controlled release over an extended period.

The drug release profiles of KFP5, KFK5, KFL5, and KFM5 demonstrated distinct triphasic or weakly triphasic patterns, influenced by polymer hydration, drug diffusion kinetics, and polymer degradation rates. These variations were further supported by pH decline and wet weight loss data, which provided insights into polymer erosion and degradation. The drug release kinetics models further highlighted the underlying mechanisms governing drug release over time.

KFK5 and KFL5 exhibited rapid drug release within the first week (37–53%), followed by a steady diffusioncontrolled phase from days 7 to 28, and a final acceleration beyond day 28. The presence of *Koliphor*^*®*^ *RH 40* and *Labrasol*^*®*^ as surfactants in KFK5 and KFL5 likely enhanced water uptake and drug solubility, resulting in a slightly higher drug release in the first week compared to KFP5. The release kinetics further supported these observations: KFP5 followed the Hixson-Crowell model, indicating that polymer erosion significantly contributed to drug release, whereas KFK5 and KFL5 followed first-order kinetics, suggesting that drug release was primarily concentration-dependent and diffusion-driven. The final phase in both formulations, occurring after day 28, aligned with a steeper pH drop and increased polymer degradation, confirming that polymer erosion played a role in the third release phase.

KFM5 exhibited the slowest initial burst release after 24 h and a moderate drug release in the first week, followed by a steady, diffusion-controlled release phase up to day 28. Unlike the other formulations, KFM5 maintained a gradual release profile throughout, with minimal fluctuations. The release kinetics followed the Higuchi model, indicating that diffusion was the dominant mechanism rather than polymer erosion. However, after day 28, a mild acceleration in drug release was observed, suggesting the onset of polymer degradation. This weaker polymer erosion effect, supported by a gradual pH decline and slower wet weight loss, suggested that KFM5 followed a weakly triphasic release pattern. While a late-stage polymer degradation-driven release phase was present, it was less pronounced compared to the other formulations.

Polymer degradation was further evaluated through pH variations and wet weight analysis. The wet weight loss trends are closely correlated with pH decline in the release medium and drug release behavior. Formulations with rapid early-stage weight loss (KFK5, KFL5) exhibited a steeper pH drop, indicative of faster polymer hydrolysis, whereas formulations with slower initial weight loss (KFM5) maintained a more stable pH before undergoing a delayed pH decline in later weeks. The degradation profile of KFM5 demonstrates that *Maisine*^*®*^ initially stabilizes the polymer matrix, delaying hydrolysis before eventual polymer erosion takes place. These findings confirm that KFP5 follows a triphasic degradation and drug release pattern, whereas KFK5, KFL5, and KFM5 exhibit triphasic or weakly triphasic degradation and release behaviors. The pH decline observed in later weeks for KFK5, KFL5, and KFM5 further validates the presence of a third-phase polymer erosion-driven drug release, distinguishing them from KFP5, which undergoes a steadier degradation process. The differences in degradation rates and drug release kinetics highlight the critical role of formulation composition, particularly the inclusion of surfactants or lipid-based excipients, in modulating polymer hydration, erosion, and drug release duration.

Moreover, the ability of KFM5 to maintain the pH of the release medium closer to physiological conditions highlights its potential for improved biocompatibility and reduced risk of local irritation, as a rapid pH drop can trigger inflammation and ocular irritation, which may compromise patient tolerability [61, 62]. This finding is further supported by the *in vitro* biocompatibility assessment results.

According to the drug release profile results, KFM5 was further been characterized for the rheological fluid behavior, predicted for the injectability parameters, and analyzed for the *in vitro* biocompatibility assessment. The rheological behavior and predicted injection force analysis provided further insights into the formulation’s injectability and clinical feasibility. The flow rate and injection force prediction showed that KFM5 could be easily administered using 19–25G needles for a more comfortable injection. While the 27G needle exhibited higher predicted injection forces, they remained within an acceptable range of below the 40 N threshold, indicating that KFM5 can be delivered using a range of needle sizes depending on the required administration method. This finding is critical, as excessive injection force can lead to patient discomfort and difficulty in administering the formulation in a clinical setting [59].

Thermal characterization using DSC revealed that *ketotifen fumarate* transitioned into an amorphous state within KFM5, as indicated by the absence of a sharp melting peak. The amorphization of the drug enhances solubility and dissolution rates, preventing potential crystallization issues that could affect long-term drug stability.

Finally, the biocompatibility assessment of KFM5 confirmed that the formulation is non-toxic to corneal epithelial cells, with cell viability remaining above 90% across all tested conditions. The live/dead cell staining further demonstrated a predominance of live cells, with minimal cytotoxicity observed. These findings validate the safety of KFM5 for ocular use, ensuring that the formulation does not induce adverse cellular responses upon administration. The combination of controlled release, pH stability, reduced burst release, and excellent biocompatibility positions KFM5 as a promising candidate for sustained subconjunctival drug delivery.

## Conclusions

This study demonstrates that the *ketotifen fumarate*-loaded *PLGA*-based ISFSI, particularly KFM5, offers notable advantages, including enhanced injectability, sustained drug release, predictable diffusion kinetics, improved polymer stability, and excellent ocular biocompatibility. These findings provide valuable insights into the potential of the ISFSI system for long-term ocular drug delivery, particularly in the treatment of chronic ocular conditions such as *allergic conjunctivitis* and other inflammatory eye diseases. The ability of KFM5 to achieve diffusion-controlled release while maintaining a stable pH and minimizing polymer degradation suggests its potential to deliver prolonged therapeutic benefits while mitigating the risks of rapid drug clearance and local inflammation. These characteristics position KFM5 as a potential candidate for further preclinical and clinical studies to evaluate its pharmacokinetics, therapeutic efficacy, and *in vivo* biocompatibility. Our future research is focused on *in vivo* ocular pharmacokinetics and long-term ocular safety to support its clinical translation for sustained ocular drug delivery.

## Supplementary Material

Supplementary Data

**Supplementary Information** The online version contains supplementary material available at https://doi.org/10.1208/s12249-025-03142-3.

## Figures and Tables

**Table I:** Composition of PLGA-based ISFSIs Loaded With 5% *Ketotifen Fumarate*

Formula	Drug conc (% w/w)	*PLGA* conc (% w/w)	DMSO conc (% w/w)	Drug release retarder	

				Type	Conc. (% w/w)

KFP5	5	30	65	-	-
KFK5	5	30	55	*Kolliphor*^®^ RH 40	10
KFL5	5	30	55	*Labrasol* ^®^	10
KFM5	5	30	55	*Maisine* ^®^	10

**Table II:** Validation of HPLC–UV Method for *Ketotifen Fumarate*-loaded ISFSI

Linearity, LOD, LOQ	R2	LOD (μg/mL)	LOQ (μg/mL)	
Ketotifen fumarate	0.9999	0.81	2.45	
System Suitability	Tailing factor (USP)	Theoretical plates (USP)	Peak area (mean ± SD)	RSD (%, *n* = 6)
Ketotifen fumarate-loaded ISFSI	0.92 ± 0.003	3080.06 ± 46.46	5207635.83 ± 28993.12	0.56
Precision	Theoretical Conc. (μg/mL)	Experimental Conc. (μg/mL)	Recovery (%)	RSD (%, *n* = 6)
Ketotifen fumarate-loaded ISFSI	100	100.43 ± 0.53	100.43 ± 0.53	0.53

**Table III:** Kinetic Model Parameters for *In Vitro* Drug Release Profiles

Factors studied	Zero-order (R^2^)	First-order		Higuchi		Hixson-Crowell		Korsmeyer-Peppas		
				
		R^2^	k_1_	R^2^	k_H_	R^2^	k_HC_	R^2^	*n*	k

KFP5	0.9624	0.9705	0.07	0.9506	18.02	0.9792	0.08	0.9082	0.57	11.11
KFK5	0.9412	0.9832	0.05	0.9811	14.39	0.9823	0.06	0.9824	0.47	15.24
KFL5	0.8647	0.9822	0.07	0.9660	16.20	0.9687	0.08	0.9192	0.60	13.14
KFM5	0.9286	0.9778	0.05	0.9798	15.91	0.9704	0.06	0.9245	0.76	6.74

**Table IV:** Predicted Flow Rate and Injection Force Values at a Shear Rate of 1500 s^−1^ Using the Power Law Equation

Needle type	The inner radius of the needle (mm)	Flow Rate (mL/min)	Injection Force (*N*)

19 G	0.69	22.09	7.43
20 G	0.603	15.00	8.46
21 G	0.51	9.08	10.00
25 G	0.26	1.20	19.61
27 G	0.21	0.63	24.28

## Data Availability

All data generated or analyzed during this study are included in this published article. Additional data are available from the corresponding author upon reasonable request.
