# Supplementary material for: Injectable PLGA-based In Situ Forming Subconjunctival Implant for Sustained Ocular Delivery of Ketotifen Fumarate: Formulation, Drug Release, and Biocompatibility Studies
Source: AAPS PharmSciTech. Author manuscript; Available in PMC 2026 Aug 21. (PMC13495649; doi:10.1208/s12249-025-03142-3)
Supplement: Supplementary file 1 [file NIHMS2192887-supplement-Supplementary_Data.docx]

# **Supplementary Data**

# **Injectable PLGA-based *in situ* forming subconjunctival implant for sustained ocular delivery of *ketotifen fumarate*: Formulation, drug release, and biocompatibility studies**

Nur Mita ^1,2^, Xuejia Kang ^1,3^, Pengyu Chen^3^, Oladiran Fasina^4^, Shenise Howard^5^, Anna-Catherine Bowden^5^, Richard J. McMullen Jr.^5,6^, Amol Suryawanshi^7^, R. Jayachandra Babu ^1,*^

^1^Department of Drug Discovery and Development, Harrison College of Pharmacy, Auburn University, AL 36849, USA

^2^Faculty of Pharmacy, Mulawarman University, Samarinda, Kalimantan Timur, 75119, Indonesia

^3^Materials Research and Education Center, Materials Engineering, Department of Mechanical Engineering, Auburn University, Auburn, AL 36849, USA

^4^Department of Biosystems Engineering, Samuel Ginn College of Engineering, Auburn University, Auburn, AL 36849, USA

^5^Department of Clinical Sciences, College of Veterinary Medicine, Auburn University, AL 36849, USA

^6^Equine Department, Vetsuisse Faculty, University of Zurich, 8057 Zurich, Switzerland

^7^Department of Population Health and Pathobiology, College of Veterinary Medicine, North Carolina State University, Raleigh, NC, USA

*Corresponding author

R. Jayachandra Babu: [ramapjb@auburn.edu](mailto:ramapjb@auburn.edu) **List of Supplementary Table and Figures**

[Table S1. The needle dimensions used in the injectability parameters prediction](#_Toc192759704)

[Figure S1. Comparison of initial burst release at 24 hours for different PLGA concentrations and various release-modifying agents](#_Toc192759705)

[Figure S2. HPLC chromatogram of *ketotifen fumarate* (100 μg/mL) compared to the diluent (blank) for baseline assessment](#_Toc192759706)

[Figure S3. Calibration curve of *ketotifen fumarate*: Plot of peak area versus concentration (μg/mL) obtained from HPLC analysis](#_Toc192759707)

Table S1. The needle dimensions used in the injectability parameters prediction

| Needle Dimension | 𝑅*n* (mm) |
| --- | --- |
| 19 Gauge | 0.69 |
| 20 Gauge | 0.60 |
| 21 Gauge | 0.51 |
| 25 Gauge | 0.26 |
| 27 Gauge | 0.21 |

Figure S1. Comparison of initial burst release at 24 hours for different PLGA concentrations and various release-modifying agents

Figure S2. HPLC chromatogram of ketotifen fumarate (100 μg/mL) compared to the diluent (blank) for baseline assessment

Figure S3. Calibration curve of ketotifen fumarate: Plot of peak area versus concentration (μg/mL) obtained from HPLC analysis
